# What is Good Training? Research to Come

**DOI:** 10.31662/jmaj.2025-0302

**Published:** 2025-08-08

**Authors:** Hiro Nakao, Osamu Nomura, Naoya Tonegawa, Mitsuru Kubota, Akira Ishiguro

**Affiliations:** 1 Department of General Pediatrics and Interdisciplinary Medicine, National Center for Child Health and Development, Tokyo, Japan; 2 Center for Postgraduate Education and Training, National Center for Child Health and Development, Tokyo, Japan; 3 Medical Education Development Center, Gifu University, Gifu, Japan

**Keywords:** work style reform, resident, patient care ownership, burnout, autonomy

## Dear Editors,

We deeply appreciate Dr Matsubara’s opinion on work style reform ^(1)^. As noted in the Letter, “autonomous and meaningful” longer work hours might have positive aspects ^(1)^, if we could determine what constitutes that kind of work and what does not. We are afraid that this judgment may be inherently hard to make, given the imbalanced relationship between faculty and residents. Research is needed on how to judge “autonomous and meaningful” works or the measurement method of personal desire to “learn more” ^(1)^. In any case, it is essential to create a work environment where residents can learn and work without relying on “autonomy and meaningfulness” as justification for long work hours, because it is unrealistic to expect all doctors to always be in excellent condition. Our past residency training was full of such justifications and victims.

Dr Matsubara introduced the Self-Determination Theory (SDT), particularly the concepts of autonomy, competence, and relatedness ^(1)^. With similar concern, we are conducting research on patient care ownership (PCO) as the key concept (papers in submission), because PCO has a measurement scale ^(2)^. PCO means a comprehensive sense of responsibility for the care of each patient and provides the basis for learning ^(2), (3), (4), (5)^. Notably, PCO was reported to have an inverse correlation with burnout ^(2)^. The PCO measurement scale includes items on autonomy ^(2)^, although the relationship between PCO and autonomy is a topic of subtle discussion ^(4)^. In short, relatedness is important in fostering PCO ^(4), (5)^ autonomy constitutes PCO ^(2)^, and PCO is necessary to achieve competence ^(4), (5)^ and is the opposite of burnout ^(2)^. As mentioned in the paper, our goal is not shorter work hours per se, but rather a training environment that enhances PCO, as well as medical knowledge and skills, while promoting trainees’ wellness ^(3)^, which we believe constitutes good training. Short work hours are just halfway to the goal.

On a final note, we have been receiving several opinions and impressions on our paper on work style reform ^(3)^. We fully agree with Dr Matsubara’s perspective that it is not only opinions and impressions that are important, but also research on medical training ^(1)^, such as research about what autonomous and meaningful work is, SDT, or PCO on medical training, and so forth. We eagerly await research to come from and into the fundamental question, “What is good training?.”

## Article Information

### Conflicts of Interest

None

### Author Contributions

Hiro Nakao, Osamu Nomura, Naoya Tonegawa, Mitsuru Kubota, and Akira Ishiguro identified the significance; Hiro Nakao and Osamu Nomura wrote the original draft; Naoya Tonegawa, Mitsuru Kubota, and Akira Ishiguro reviewed and edited the draft; and all authors approved the final manuscript.

### Approval by Institutional Review Board (IRB)

Not applicable.

### Data Availability Statement

Not applicable.
